# Evaluation of diosgenin content from eleven different Indian varieties of fenugreek and fenugreek leaf powder fortified bread

**DOI:** 10.1007/s13197-021-04967-z

**Published:** 2021-01-24

**Authors:** Mahadevappa Paramesha, Nagbhushan Priyanka, Kasar Crassina, Nandini Prasad Shetty

**Affiliations:** 1 Plant Cell Biotechnology Department, CSIR – Central Food Technological Research Institute, Mysore, 570 020 India; 2 Flour Milling, Baking and Confectionery Technology, CSIR – Central Food Technological Research Institute, Mysore, 570 020 India; 3 Department of Food Technology, Davangere University, Davangere, Karnataka 577007 India

**Keywords:** Fenugreek, Diosgenin, Phenolics, Flavonoids, Bread

## Abstract

The present study designed to establish the diosgenin profile from eleven different Indian varieties of fenugreek, and subsequently develop powder from the best stage and variety to prepare diosgenin fortified bread. The seeds, sprouts and leaves of different fenugreek varieties were analyzed for the diosgenin using HPLC. The content of phenolics and flavonoids also estimated and assessed for its antioxidant capacity using phosphomolybdate, DPPH and FRAP. Among eleven varieties screened for diosgenin and the other compounds, tender leaf of Kasuri methi (KS) variety showed the highest content of diosgenin (466.89 ± 0.32 mg/100 g FW), phenolics (58.54 ± 2.70 mg/100 g FW) and flavonoids (1104.16 ± 43.70 mg/100 g FW) followed by Pusa Early Bunching (PEB) (444.18 ± 0.36 mg/100 g FW) and Early Bunching (EB) (409.45 ± 0.42 mg/100 g FW). Among three stages, seeds found to be a better total antioxidant, DPPH scavenging, and reducing ability. Further, based on the results, bread fortified with 1.5% KS leaf powder is found to be optimal which also has significant diosgenin content (268.91 mg/100 g DW). And the effect of KS leaf powder on amylograph and farinograph characteristics of wheat flour and quality characteristics of bread showed promising results of acceptance.

## Introduction

Fenugreek (*Trigonella foenum-graecum L*.) is well-known for its medicinal property all over the world (Ahmad et al. 2016). Fenugreek is with trifoliate, branched stem, roots bearing nodules, white flowers, with golden yellow seeds (Khare 2004). It has been used in cooking to enhance the flavour, colour and to modify the texture of the food materials. It is also a well documented oldest medicinal plant, traditionally used in India to treat a variety of diseases since long back (Khare 2004; Ahmad et al. 2016). The tender raw leaves of fenugreek were used in salad and cooked dishes in India (Sarwar et al. 2020). Fenugreek seeds and leaves are rich in fibre, protein, β-carotene, vitamins, minerals, gums along with alkaloids, flavonoids, steroidal sapogenins, diosgenin, trigocoumarin, nicotinic acid, trimethyl coumarin and trigonelline (Khare 2004; Naidu et al. 2011; Ahmad et al. 2016; Sarwar et al. 2020). Fenugreek possesses several medicinal properties like antimicrobial, antidiabetic, hypocholesterolemic, chemopreventive, gastroprotective, anti-inflammatory, antipyretic, hepatoprotective, antioxidant, anticancer and good lactating aid in weaning mother etc., (Khare 2004; Naidu et al. 2011; Ahmad et al. 2016; Sarwar et al. 2020). Diosgenin present in fenugreek has paved way to many researchers due to its medicinal properties.

Diosgenin (25R-spirost-en-3β-ol), a plant derived steroidal sapogenin and found in many plants such as fenugreek, china root (*Smilax china*), etc. (Deshpande and Bhalsing 2014). Diosgenin used extensively as an essential compound for the production of sex hormones, oral contraceptives pills and steroidal drugs due to its extensive pharmaceutical applications (Deshpande and Bhalsing 2014). Diosgenin found to be effective against disorders like diabetes, hyperlipidemia, different types of cancer, osteoporosis, cardiovascular diseases, skin diseases, oxidative stress, inflammation and neurological diseases (Deshpande and Bhalsing 2014; Dangi et al. 2014; Cai et al. 2020). However, due to its low solubility in water and poor bioavailability, it is hard to explore the medicinal property of diosgenin (Cai et al. 2020). Therefore, food formulation may be an excellent alternative to provide diosgenin to the layman in a cost-effective manner. The purpose of the current study is to establish knowledge on the changes in diosgenin, total phenolics, flavonoids and antioxidant quality of fenugreek extracts from different varieties at different growth stages. This information would help in deciding the best variety to cultivate and also to use the best stage for the development of functional food. Therefore, the present work is primarily focused on the evaluation of diosgenin content using HPLC method. Further, the total phenolics and flavonoids were also assessed in eleven different Indian fenugreeks varieties at different stages. Subsequently, the leaf which was found to retain the highest amount of diosgenin was further processed into powder for fortifying bread and also studied the effect of fenugreek leaf powder (FLP) on the bread-making characteristics of wheat flour.

## Materials and methods

### Plant materials

Purchased the mature seeds of fenugreek varieties Maher-1 (MH-1), Early Bunching (EB); Kasuri methi (KS); PEB (Pusa Early Bunching); MG; CO-1; CO-2; Lambel-1(LA-1); Local (LO); RMT-303 and RMT-305 from Tamil Nadu Agriculture University, Coimbatore and local market of Mysore, India. The plants (5–6 leaf staged) were grown in greenhouse of the Plant Cell Biotechnology Department, CFTRI, Mysuru, Karnataka. The seeds were soaked in distilled water for overnight. Then tied the one part of soaked seeds in a cloth for the emergence of sprouts and another part is sowed in sterile peat and grown in a greenhouse for further studies.

### Estimation of total phenolics (TPC), flavonoids content (TFC) and antioxidant assays

The extraction efficiency of antioxidant compounds in 80% ethanol was tested against different parts of fenugreeks following an earlier reported method (Kumar et al. 2020). Briefly, extracted about 2.5 g of sample (seed/sprouts/leaf) with 50 ml of 80% ethanol by using mortar and pestle, using gyro rotary shaker the extraction was shaken at 120 rpm for 30 min and centrifuged at 10,000 rpm for 10 min. The pellet was collected and re-extracted, and both the supernatants were pooled and stored in amber tubes to avoid light interference. The total phenolic and flavonoids content of different varieties of fenugreek extract determined by the method described by Kumar et al. (2020) respectively. The concentration of TPC and TFC was expressed as mg gallic acid equivalent (GAEq.) 100 g and mg rutin equivalent (REq.) 100 g FW of the sample. Further, the methanolic extract of all the samples were subjected for Total antioxidant capacity (Kumar et al. 2020), 2,2-diphenylpicrylhydrazyl (DPPH) radical quenching ability (Kumar et al. 2020) and ferric ion reducing power assay (Kumar et al. 2020). Ascorbic acid was used as a standard, and absorbance was read using a double-beam spectrophotometer (UV-160 A, Shimadzu Corporation, Kyoto, Japan).

### Extraction and high-performance liquid chromatography (HPLC) characterization of diosgenin

With small modifications, diosgenin was extracted by the process of hydrolysis with ethanolic sulfuric acid as described by Chaudhary et al. (2018). Briefly, 1 g of oven-dried (45 °C) samples (seed, sprouts, tender leaf and bread) was macerated with mortar and pestle using 15 ml of 2.5 M ethanolic sulfuric acid. The macerate was refluxed for 4 h at 73–74 °C, cooled, filtered through Whatman No.1 filter paper. The obtained filtrate pH was adjusted to 7.0 with 10 M NaOH then extracted with n-hexane (3 × 15 ml). The n-hexane extract phase was evaporated using rotavapor (Hei-VAP Advantage, Heidolph Instrument GmbH & Co. KG, Schwabach, Germany). The residue obtained was dissolved in 1 ml of HPLC grade acetonitrile and water (9:1). The samples were filtered 0.45 µm filter and used for diosgenin analysis by HPLC connected with C18 Luna, (250 × 4.6 mm, 12 nm, 5 µm) column. An isocratic separation with acetonitrile: water (90:10 v/v) at a flow rate of 1 ml/min at 35 °C and diosgenin was detected at 194 nm. The standard diosgenin compound (Sigma-Aldrich, Bangalore, India) used to identify and quantify the diosgenin from the sample extracts.

### Preparation of wheat flour (WF): fenugreek leaf powder (FLP) blends

The leaf of Kasuri methi, which was found to contain the highest amount of diosgenin, was dried in a hot air oven at 45 °C for 4 h. The dried leaves were made into powder of particle size of 150 µm using a mixer grinder (Amaze 780 W, Inalsa Appliances, India) to obtain fenugreek leaf powder (FLP) and it will be easy to blend with wheat flour. Further, the FLP was thoroughly subjected for the estimation of TPC, TFC and Diosogenin content before blending with wheat flour. Blends of WF: FLP were prepared by mixing wheat flour with varying ratio of FLP (0%, 0.5%, 1%, 1.5%, 2%) by dry mixing in a planetary mixer for 5 min for uniform blending. The blends were packed in airtight polypropylene covers and stored in the refrigerator (− 4 °C) for rheological and baking experiments.

### Wheat flour quality and rheological characteristics of blends

The chemical characteristics of wheat flour namely moisture (method 44–16), protein (method 46–10), ash (method 08–01), dry gluten (method 38–10), Zeleny’s sedimentation value (method 56-61A) and falling number (method 56-81b), were determined using standard methods of AACC (2000).

Farinograph was used for studying the mixing profile of wheat flour as influenced by the addition of FLP (Brabender Farinograph, Model No. 810108004, Duisburg, Germany) following a standard method (54–21) of AACC (2000). Amylograph represents the pasting properties of wheat flour, and blends were determined according to AACC (2000) method (22–10) using the Micro-Viscoamylograph (Model no.803201, Brabender, Duisburg, Germany).

### Test baking of bread

Used the following formulations for the preparation of bread: wheat flour (100 g), fenugreek leaf powder (0.5, 1.0, 1.5 and 2.0% on flour basis) separately; 2 g compressed yeast purchased from Tower brand, AB, Mauri, India Pvt. Ltd., Chennai, India, common salt (1.5 g), 12 g of hydrogenated fat (Bunge India Pvt Ltd., Mumbai, India), and water (optimum water absorption as determined with the farinograph). Bread in quadruplicate was prepared by mixing the ingredients in a Hobart mixer (Model N-50, Hobart, GmbH, Offenburg, Germany) with a flat blade for 4 min at 61 rpm. The dough was fermented in a chamber maintained at 30 °C and 75% relative humidity (RH) for 90 min, remixed, rounded, and again fermented for 25 min, moulded, proofed for 55 min at 30 °C, 85% RH, and baked for 25 min at 220 °C. The bread was cooled, packed and stored at room temperature for 24 h for physical and sensory analyses, whereas for later use for chemical analyses, the bread was stored at − 20 °C.

### Physical parameters of bread

Bread weight was recorded, and volume was measured by rapeseed displacement method (Pierce and Walker 1987). Crumb firmness (method 74–09) was measured according to AACC (2000) procedure using a texture analyzer (Model TA-HDi, Stable Microsystems, Surrey, UK) under the following conditions: sample thickness, 25 mm; load cell, 10 kg; plunger diameter, 36 mm; and plunger speed, 100 mm/min. Crumb firmness, in terms of force (N) required for 25% compression, was measured. The control bread and bread with different levels of FLP were also analyzed for moisture content (method 44–15) according to the standard methods (AACC, 2000). Values reported were averages of three determinations. Colour measurement values of control and experimental bread samples were measured in terms of lightness (L) and colour (+ a: red, − a: green, + b: yellow, − b: blue) using hunter lab colorimeter (colour measuring lab scan XE system, USA). A standard whiteboard made from barium sulphate (100% reflectance) was used as a perfectly white object for setting the instrument with D illuminant. Samples were placed in a sample holder, and the reflectance was recorded for the wavelength ranging from 300 to 800 nm. Average of three readings was reported.

### Statistical analysis

The data in the manuscript has been presented in mean ± SD of triplicates. Statistical analysis was performed with one-way analysis of variance (ANOVA) followed by Turkey’s post-test. *P* < 0.05 was considered as statistically significant. Duncan’s New Multiple Range Test was performed for the farinograph characteristics, physical parameters and moisture content of bread (Duncan 1955). A significance level of *p* < 0.05 was adopted for the comparison.

## Results and discussion

### Total phenolic, flavonoids and antioxidant properties

In recent years, the role of bioactive components like phenolics, flavonoids, saponins, alkaloids etc. from cereals, pulses, fruits and vegetables are gaining greater attention and these found to be vital resources for the formulation of drugs/food to treat the diseases (Nambiar et al. 2015). The TPC and TFC of 11 varieties of fenugreek were provided in Table 1. Among three different samples, seeds were found to be rich in TPC ranging from 47 mg/100 g FW (CO-2) to 215 mg/100 g FW (RMT-305). In sprouts (EB variety 67 mg/100 g FW) and leaf (KS variety 58.5 mg/100 g FW) were recorded. However, the seeds (CO-1, 1120.8 mg/100 g FW) and leaf (KS, 1104.1 mg/100 g FW) of fenugreek were found to be rich in flavonoids when compared to sprouts (EB, 340 mg/100 g FW). Furthermore, the results clearly showed a significant increase in flavonoid content from seeds (KS, 666.25 mg/100 g FW) to leaf (KS, 1104.16 mg/100 g FW).

**Table 1:** Total phenolic and flavonoids content in different samples

Fenugreek varieties	Total phenolic content (in mg/100 g FW )			Total flavonoids content (in mg/100 g FW)		
	Seed	Sprout	Leaf	Seed	Sprout	Leaf
MH1	68.19 ± 9.21	64.65 ± 3.38	52.84 ± 3.96	687.50 ± 70.38	305.83 ± 30.00	943.33 ± 20.96
EB	81.87 ± 3.08	67.08 ± 8.00	29.93 ± 9.83	640.83 ± 36.14	340.41 ± 11.34	475.41 ± 12.14
KS	98.81 ± 9.21	49.30 ± 8.75	58.54 ± 2.70	666.25 ± 35	206.25 ± 9.43	1104.16 ± 43.70
PEB	75.00 ± 4.56	52.36 ± 4.57	54.09 ± 8.14	563.33 ± 29.95	319.16 ± 27.79	922.50 ± 35
MG	87.84 ± 16.69	57.29 ± 5.95	25.97 ± 4.30	564.58 ± 33.94	304.58 ± 42.30	558.33 ± 32.53
LO	69.72 ± 4.79	51.38 ± 7.95	55.13 ± 5.20	531.25 ± 35	297.50 ± 34.39	897.91 ± 30.00
CO-1	115.83 ± 12.44	38.33 ± 1.80	15.20 ± 1.85	1120.83 ± 52.20	246.66 ± 6.41	149.58 ± 26.40
CO-2	47.91 ± 3.66	60.06 ± 9.88	23.12 ± 2.95	554.58 ± 51.61	312.91 ± 31.72	60.416 ± 2.60
LA-1	53.33 ± 4.92	59.23 ± 13.96	45.00 ± 1.45	474.58 ± 55.35	246.25 ± 16.25	400.83 ± 28.89
RMT-303	55.13 ± 4.91	38.33 ± 3.61	32.15 ± 1.14	830.83 ± 59.61	230.41 ± 27.53	286.25 ± 7.80
RMT-305	215.55 ± 7.01	36.80 ± 1.82	35.20 ± 3.00	558.33 ± 52.91	211.25 ± 14.08	199.16 ± 6.41
Dry KS Leaf^#^	622.12 ± 7**(DW)	–	–	101.43 ± 0.1**(DW)	–	–
Bread (2%)	32.89 ± 1.2*(DW)	–	–	1.37 ± 0.1*(DW)	–	–

All the values are represented its mean ± SD of three replicates

The *P value < 0.05 was considered statistically significant

^#^The selected sample was oven dried at 45 °C and conducted the estimation of TPC and TFC

**Fig. 1:**
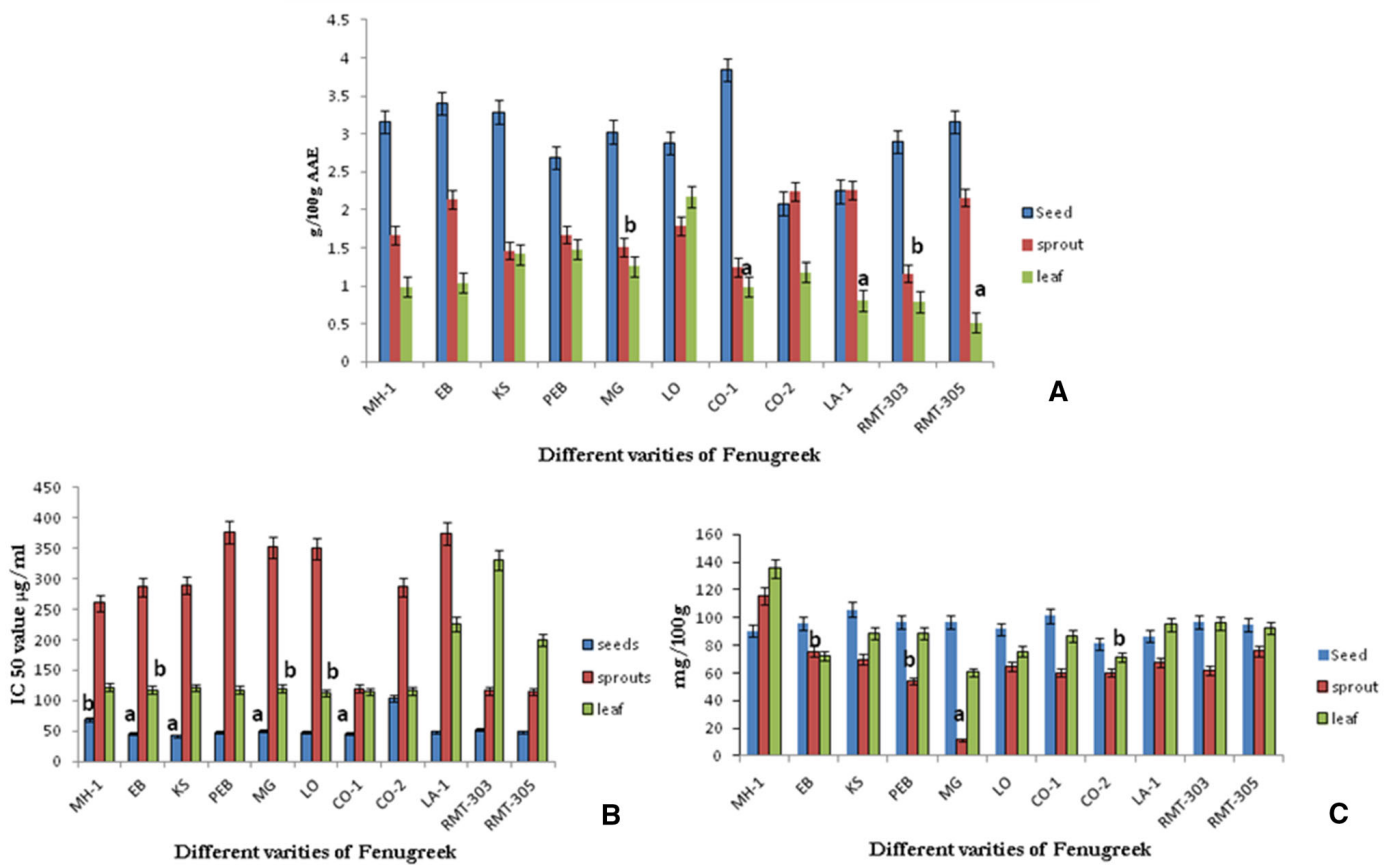
Effect of fenugreek extracts (seeds, sprouts and leaf) on different Antioxidant assays **a** Total antioxidant assay, **b** DPPH scavenging activity (IC_50_) and **c** Ferric reducing antioxidant power (FRAP). Values are means ± standard deviation (n = 3). The *P* value < 0.05 was considered statistically significant

Highly reactive free radicals and oxygen species were present in biological systems from a wide variety of sources. These free radicals may oxidize nucleic acids, proteins, lipids and can initiate degenerative diseases (Ortuno et al. 1998; Paramesha et al. 2011; Rao et al. 2011; Nambiar et al. 2015). Several clinical studies were suggesting that the antioxidants compounds in grains, oilseeds, fruits, leaf vegetables, tea and red wine are the main factors in reducing the incidence of chronic diseases including heart disease and some cancers (Li et al. 2014). Antioxidant compounds scavenge free radicals and thus inhibit the oxidative mechanisms that lead to degenerative diseases (Paramesha et al. 2011; Rao et al. 2011; Li et al. 2014; Nambiar et al. 2015; Kumar et al. 2020). The total antioxidant capacity was calculated based on the reduction ability of fenugreek extracts from Mo(VI) to Mo(V) which is readable at spectrometrically at 695 nm (Paramesha et al. 2011; Nambiar et al. 2015), and results were represented as Fig. 1A. Among three samples, seeds showed the highest retention of antioxidant compounds (3.84 g/100 g FW, CO-1) followed by sprouts (LA-1 2.26 g/100 g FW) and leaf (LO, 2.17 g/100 g FW) extracts. DPPH is a rapid and widely accepted method for its reliable and reproducibility with technically simple (Kumar et al. 2020). The method is based on the ability of extracts to reduce the purple coloured DPPH radical to colourless, which will be read at 517 nm. Results (Fig. 1B) showed that the seeds have the significant DPPH radical quenching ability with EC50 ranging from 42 μg/mg (KS) to 104 μg/mg (CO-2). Whereas, in sprouts (114 μg/mg–376 μg/mg) and leaf (112 μg/mg–330 μg/mg) required a higher concentration. The reductants present in the given extract give the reducing ability, and such extracts exhibit the antioxidative potential by breaking the free radical chain, by donating a hydrogen atom (Duh 1998; Paramesha et al. 2011). The dose-dependent ferric ion reducing ability was observed in the extract. The reductants present in the extracts found to be effective inhibitors of lipid peroxides which leads to the injury of the liver (Velavan et al. 2007). The reductants of extract reduce the Fe3 + ion in the ferricyanide complex to the ferrous form, which is read at 700 nm (Velavan et al. 2007; Paramesha et al. 2011). Among three different samples, the seed extracts showed better ferric ion reducing capacity, and it is followed by leaf and sprouts, respectively (Fig. 1c). The antioxidant capacity of different samples was attributed to the total phenolic and flavonoids content present in the extracts, and many researchers also found similar results for different food and medicinal plants (Velavan et al. 2007; Paramesha et al. 2011; Rao et al. 2011; Li et al. 2014; Nambiar et al. 2015; Kumar et al. 2020).

### Estimation of Diosgenin by HPLC method

Diosgenin profile was constructed for different samples (seeds, sprouts and tender leaf) of different varieties of fenugreek and FLP fortified bread using HPLC. The results were represented in Table 2. Diosgenin content was found in the range between 200 and 480 mg/100 g FW quantified with standard diosgenin. Among the three samples, tender leaf found to be a better source for diosgenin followed by seeds and sprouts. Among 11 varieties, the KS tender leaf (466.89 ± 0.32 mg/100 g FW) showed the highest diosgenin, and lowest was recorded in the leaf of LA-1 (187.33 ± 0.54 mg/100 g FW). Further, seeds found to be a better source for diosgenin compared to sprouts. The results of HPLC data of diosgenin showed that the tender leaf of fenugreek is the best source for the diosgenin, and Ortuno et al. (1998) recorded similar results. Dangi et al. (2014) reported the seeds of *T. foenum-graecum* L. showed highest diosgenin compare to other aerial parts of the plant. However, in our report, it is established that the tender leaves are the best source of diosgenin compared to the seeds and sprouts. Therefore, our results support the usage of raw tender leaf fortified salads and cooked food to get good diosgenin content. The FLP fortified bread also showed the excellent retention of diosgenin content even after the baking at high temperature. Among four different concentrations, 1.5% FLP containing bread marked the highest content of diosgenin (298.7 ± 5 mg/100 g FW). Many researchers reported the medicinal importance of diosgenin on many diseases such as pathologies, including diabetes, hyperlipidemia, cancer, cardiovascular disease, oxidative stress, and inflammation etc. (Pari et al. 2012; Deshpande and Bhalsing 2014; Dangi et al. 2014; Cai et al. 2020).

**Table 2:** HPLC profiling of diosgenin content in different samples and KS-FLP fortified Bread

Sample Name	Concentration of diosgenin in mg/100 g			Diosgenin content in bread (mg/100 g)
Variety	Seed	Sprouts	Leaf	
MH-1	287.8 ± 0.5*	249.2 ± 0.3*	334.2 ± 0.5*	245.7 ± 3 (0.5%)
EB	269.3 ± 0.4	242.2 ± 0.5	409.5 ± 0.4**	258.2 ± 1(1.0%)
KS	286.8 ± 0.6*	229.8 ± 0.4	466.9 ± 0.3**	298.7 ± 5** (1.5%)
PEB	291.3 ± 0.5**	245.7 ± 0.5*	444.2 ± 0.4**	282.7 ± 8**(2.0%)
MG	277.8 ± 0.1	237.2 ± 0.3	395 ± 0.3*	
LO	284.6 ± 0.6	230.9 ± 0.2	400 ± 0.6*	
CO-1	326.2 ± 0.3**	249.6 ± 0.4	217.8 ± 0.5	
CO-2	305.6 ± 0.7**	260.8 ± 0.2**	199.2 ± 0.4	
LA-1	262.7 ± 0.6	256.1 ± 0.4*	187.3 ± 0.5	
RMT-303	285.9 ± 0.5*	273.3 ± 0.4**	205.2 ± 0.7	
RMT-305	288.3 ± 0.9**	253 ± 0.6*	207.2 ± 0.4	
Dry KS Leaf			326.1 ± 0.8*	

Values are means ± standard deviation (n = 3), The concentration of FLP in the bread mentioned in the bracket

The *P value < 0.05 was considered statistically significant

## Fenugreek leaf powder fortified bread

### Effect of FLP on amylograph and farinograph characteristics of wheat flour

Wheat flour used for the test of baking bread was of the medium strong type having 11.5% moisture, 0.3% ash, 10.2% protein, 8.9% dry gluten, 27 ml Zeleny’s sedimentation value, and 566 s Hagberg falling number. The moisture content of FLP used in the study had a moisture content of 7.4%. The mixing profile of wheat flour as influenced by varying levels of FLP is presented in Table 3, Water absorption, the amount of water required for the dough to have a desired definite consistency. As expected, FLP has increased the water absorption capacity of wheat flour; it increased from 60.5% to 63.8% as the level of FLP increased from 0 to 2%. Similarly, dough development time (DT) which relates to the time taken for the dough to reach the point of greatest torque also increased with an increasing amount of FLP. The highest DT value (6.0 min) was observed where wheat flour was replaced with FLP at 2%. The increasing trend indicates delay in hydration and mixing time of flour particles for optimum dough development. Mixing tolerance index (MTI), denotes the elasticity of the dough and is inversely proportional to the strength of the dough; higher values indicate less tolerance for mixing. No change in MTI value was observed when wheat flour was substituted with 0.5% of FLP however it increased from 18 to 60 BU as FLP increased from 1 to 2%. This may be attributed to the presence of fibre and other phytonutrients in FLP. In general, in the present study, only a low effect of the FLP on the viscoelastic behaviour of wheat flour was observed. This is desirable as it may indicate minor changes in bread, making the performance of wheat flour having different levels of FLP. The effect of fenugreek leaf powder on rheological characteristics has not been reported yet. Indrani et al. (2010) reported that replacement of wheat flour with fenugreek seed powder from 0 to 7.5% increased the farinograph water absorption from 61.8 to 72.2% and dough development time from 3.8 to 4.8 min, owing to it to the presence of gums and fibres in fenugreek seed. Similarly, Chauhan and Sharma (2000) also observed an increase in water absorption with increasing level of debittered fenugreek seed powder with no effect on the dough development time and stability up to 4.5% level of replacement.

**Table 3:** Farinograph characteristics of wheat flour as influenced by fenugreek leaf powder

Parameters	Control	FLP (%)			
		0.5	1.0	1.5	2.0
Water absorption capacity, WA (%)	60.5 ± 0.25^a^	62 ± 0.29^b^	62.7 ± 0.50^b^	63.5 ± 1.15^c^	63.8 ± 0.4^d^
Dough development time, DT (min)	1.5 ± 0.0^a^	1.9 ± 0.43^a^	3.5 ± 1.0^b^	5.3 ± 0.01^c^	5.3 ± 0.25^c^
Mixing tolerance index, MTI (BU)	18 ± 1.35^a^	18 ± 2.40^a^	24 ± 2.28^a^	53 ± 4.25^b^	60 ± 3.37^c^

Values are means ± standard deviation (n = 3), Values in the same column with different superscript letters are significantly different at p < 0.05

*FLP* Fenugreek leaf powder; *WA* Water absorption; *DT* Dough development time; *MTI* Mixing tolerance index; *BU* Brabender unit

**Fig. 2:**
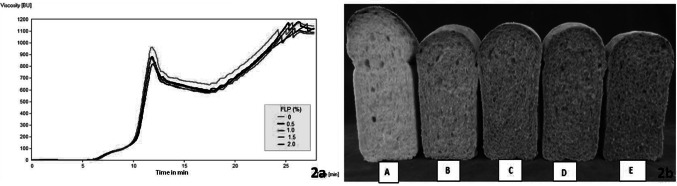
**a** Effect of fenugreek leaf powder (FLP) on amylograph characteristics of wheat flour. **b.** Photograph of breads with varying levels of fenugreek leaf powder (FLP). **A **Control (0% FLP), **b** 0.5% FLP, **c** 1% FLP, D-1.5% FLP, 2% FLP

Figure 2a represents the pasting properties of wheat flour with 0 to 2% levels of FLP. The gelatinization temperature for wheat flour with 0%, 0.5%, 1%, 1.5% and 2% FLP were in the range of 60.1–62 °C. This indicates that the minimum temperature required for the starch to swell, form a paste and get cooked is increased slightly with the presence of FLP. The peak viscosity is the highest viscosity the gelatinized starch can attain and represents the ability of the starch granules to swell freely before their physical breakdown. The peak viscosity of wheat flour decreased from 964 to 820 BU as the level of FLP increased from 0 to 2% of FLP. Our observation is in line with previous studies by Dachana et al. (2010) on quality characteristics of cookies with 0 to 15% of *Moringa oleifera* leaf powder replacement of wheat flour which also observed a decrease in the peak viscosity from 853 to 623 BU. Similar work by Sharma et al. (2013) also noted a decrease in peak viscosity from 1098 to 876 BU with 2.5–7.5% of *Tinospora cordifolia* leaf powder*. *In general, the decrease in viscosity of wheat flour with the addition of leaf powders lead to a decrease of starch and less water available for initial swelling affecting the viscosity parameters. Substitution of wheat flour with FLP decreased the setback values which indicate that retrogradation of gelatinized starch granules. The setback value of wheat flour was 1142 BU, while it was in the range of 1126–1077 BU for wheat flour with 0.5 to 2% of FLP. This is basically due to dilution of amylose fraction of starch which is known for higher retrogradation in cooked products. In contrast to our observation with FLP on pasting properties of wheat flour, a study by Brennan et al. (2006) showed that fenugreek seed powder has an increasing effect on peak viscosity, cold paste viscosity and setback attributing mainly to the galactomannan present in fenugreek seed. Further, Funami et al. (2005) described that non-ionic polysaccharides could help amylose gelation and hence increase the setback value of starch material.

### Quality characteristics of bread

The moisture content of control bread was 29.87%, and bread with FLP was in the range of 32.92%–36.46% (Table 4). The difference in the moisture content is due to the different water uptake during the mixing stage to obtain optimum dough consistency. The photograph of bread with varying amount of FLP is shown in Fig. 2b. The specific volume in case of control was 4.05 cm3/g, whereas it showed a decreasing trend with the increasing level of FLP from 0.5 to 2%. This observation is also indicated in Fig. 2b. However, the bread with 0.5–1.5% FLP does not vary significantly (*p* < 0.05) in terms of specific volume. The crumb firmness, a measure of the texture of control bread was 6.97 N, and it was in the range of 8.08–10.01 N for bread with FLP. The results indicate that substitution of wheat flour with FLP had a slight stiffening effect on the bread crumb. In the case of colour measurements, as expected, the control bread was lightest with *L* value of 66.88, whereas the bread with FLP became darker in colour (57.08–46.25). Redness (+ *a*) also decreased with an increasing amount of FLP in bread. In contrary, yellowness (+ *b*) for control was 11.97, but it increased from 16.37 to 18.28 for the bread with FLP of 0.5 to 2%. It can be stated that the addition of FLP into bread formulation at levels of 0.5%–2% does not affect adversely the physical parameters, namely volume and texture of the bread except for the colour. Based on the yellow color and strong pungent flavor that FLP imparts on bread, it is observed that adding FLP at 1.5% is optimal where good retention of diosgenin (298.7 ± 5 mg/100 g) is also seen. Study on the use of fenugreek leaf powder in bread is rare. Considering the fact that fenugreek leaf is often used as condiments in many food products like traditional Asian flat bread, extruded snacks and savouries; incorporation of fenugreek leaf powder as a functional health ingredient in bread may as well be accepted.

**Table 4:** Physical characteristics of bread as influenced by fenugreek leaf powder (FLP)

FLP (%)	Moisture content (%)	Specific volume (cm^3^/g)	Texture (N)	L	a*	b*
0	29.87 ± 0.11^a^	4.05 ± 0.02^c^	6.97 ± 0.55^a^	66.88 ± 1.98^d^	10.93 ± 0.15^c^	11.97 ± 0.29^a^
0.5	32.92 ± 0.30^ab^	3.65 ± 0.06^b^	8.08 ± 0.52^ab^	57.08 ± 0.75^c^	− 1.36 ± 0.23^a^	16.37 ± 0.13^b^
1.0	34.92 ± 0.30^b^	3.62 ± 0.04^b^	8.79 ± 1.12^bc^	51.48 ± 1.02^b^	− 1.32 ± 0.08^a^	16.83 ± 0.23^b^
1.5	36.15 ± 3.36^c^	3.58 ± 0.03^b^	8.71 ± 2.34^bc^	49.93 ± 0.72^b^	− 1.41 ± 0.07^a^	17.45 ± 0.23^c^
2.0	36.46 ± 0.21^b^	3.45 ± 0.04^a^	10.01 ± 1.67^c^	46.25 ± 0.78^a^	− 0.97 ± 0.08^b^	18.28 ± 0.16^d^

Values are means ± standard deviation (n = 3), Values in the same column with different superscript letters are significantly different at *p* < *0.05*

*FLP* Fenugreek leaf powder, *N*- Newton, *L* Lightness/darkness; ± a: red/green; ± b: yellow/blue

## Conclusion

Among the different varieties screened, the diosgenin content was higher in KS variety with higher amount of phenolic and flavonoids also. This study suggests that the optimum metabolite and antioxidant potential in early staged leaves of fenugreek when compared to sprouts. The seeds also showed higher antioxidant activity and metabolite; however, the best activity was seen in the tender leaves of KS variety of the plant for the accumulation of secondary metabolites and pharmaceutical activity**.**

## Data Availability

All data generated or analysed during this study are included in this published article.

## Abbreviations

AACC: American association of cereal chemists
ANOVA: Analysis of variance
DPPH: 2,2-Diphenyl-1-picrylhydrazyl
DT: Dough development time
EB: Early Bunching
EC50: Effective Concentration 50
FLP: Fenugreek Leaf Powder
FRAP: Ferric Reducing Antioxidant Power
GAEq: Gallic acid equivalent
HPLC: High performance liquid chromatography
KS: Kasuri methi
LA-1: Lambel-1
LO: Local variety
MH-1: Maher-1
MTI: Mixing Tolerance Index
PEB: Pusa Early Bunching
REq.: Rutin equivalent
RH: Relative Humidity
TFC: Total Flavonoids Content
TPC: Total Phenolics Content
WF: Wheat Flour
